# CO_2_ Mineralization and Utilization using Steel Slag for Establishing a Waste-to-Resource Supply Chain

**DOI:** 10.1038/s41598-017-17648-9

**Published:** 2017-12-08

**Authors:** Shu-Yuan Pan, Tai-Chun Chung, Chang-Ching Ho, Chin-Jen Hou, Yi-Hung Chen, Pen-Chi Chiang

**Affiliations:** 1 Graduate Institute of Environmental Engineering, National Taiwan University, Taipei, 10673 Taiwan; 2 Carbon Cycle Research Center, National Taiwan University, Taipei, 10674 Taiwan; 3 Tung Ho Steel Enterprise Corporation, Miaoli, 368 Taiwan; 4 Department of Chemical Engineering and Biotechnology, National Taipei University of Technology, Taipei, 10608 Taiwan

## Abstract

Both steelmaking via an electric arc furnace and manufacturing of portland cement are energy-intensive and resource-exploiting processes, with great amounts of carbon dioxide (CO_2_) emission and alkaline solid waste generation. In fact, most CO_2_ capture and storage technologies are currently too expensive to be widely applied in industries. Moreover, proper stabilization prior to utilization of electric arc furnace slag are still challenging due to its high alkalinity, heavy metal leaching potentials and volume instability. Here we deploy an integrated approach to mineralizing flue gas CO_2_ using electric arc furnace slag while utilizing the reacted product as supplementary cementitious materials to establish a waste-to-resource supply chain toward a circular economy. We found that the flue gas CO_2_ was rapidly mineralized into calcite precipitates using electric arc furnace slag. The carbonated slag can be successfully utilized as green construction materials in blended cement mortar. By this modulus, the global CO_2_ reduction potential using iron and steel slags was estimated to be ~138 million tons per year.

## Introduction

Carbon dioxide (CO_2_) mineralization and utilization has been considered one of the imperative strategies on combating climate change and global warming^1,2^. It is noted that whilst CO_2_ capture and storage (CCS) may be an important economic incentive for some early CCS projects, CO_2_ utilization via chemical conversion may prove to be a costly distraction, financially and politically, from the real task of mitigation^3^. The main contributors to global CO_2_ emissions from industrial sectors are the steelmaking and cement industries. Both steelmaking via electric arc furnace and manufacturing of portland cement are energy-intensive and resource-exploiting processes^4^, with great amounts of CO_2_ and alkaline solid wastes, such as electric arc furnace reducing slag (EAFRS), released into the environment. In particular, stabilization and utilization of the alkaline solid wastes are critical concerns at steelmaking industries^5^.

World Steel Association estimates that over 400 million tonnes of iron and steel slags is produced each year^6^. Among various types of steel slags, basic oxygen furnace and electric arc furnace slags are the major by-products worldwide in terms of annual production quantities. In our previous research, we have evaluated the performance of CO_2_ mineralization and utilization using basic oxygen furnace slag^7^. The results indicate that the reacted basic oxygen furnace slag could be suitably utilized as supplementary cementitious materials in cement mortars. Compared to other steel slags such as basic oxygen furnace slag, the utilization of electric arc furnace slag is relatively limited. From the viewpoint of chemical compositions, EAFRS could potentially become raw materials for partial substitution in clinker or as an aggregate in concrete once it is properly stabilized^8^. In general, EAFRS has been found to be rich in CaO (45.0–50.0%), with minor phases of SiO_2_ (15.0–30.0%), Al_2_O_3_ (3.0–20.0%), and MgO (5.0–15.0%), and trace phases of MnO, Al_2_O_3_ and SO_3_ at percentages ranging from 0.2% to 5.0%^9^. The cementitious and pozzolanic characteristics of fresh EAFRS have been extensively studied in the literature to evaluate its potential for use in construction industry^10^. However, several barriers, including heavy metal leaching potentials and volume instability, have hindered its widespread use due to the presence of active components such as free lime (CaO_*f*_).

These active components in EAFRS can be easily reacted with CO_2_ via an accelerated carbonation reaction^11^, which is similar to the natural weathering processes. Since the accelerated carbonation is a diffusion control reaction, several innovative methods such as high-gravity carbonation^12,13^ have been proposed to enhance the CO_2_ mineralization efficiency and capacity. Through the reaction, the CO_2_ will convert to stable and insoluble carbonates (e.g., CaCO_3_), which are rarely released afterward^14,15^. Therefore, it can be used as an integrated approach to mineralizing CO_2_ in the flue gas while stabilizing the EAFRS. Moreover, due to its similar physico-chemical properties to Portland cement, the carbonated EAFRS product might be used as supplementary cementitious materials in blended cement^10^. However, to the best of our knowledge, few research has been focused on both the efficiency of CO_2_ mineralization using EARFS, as well as on the utilization of carbonated EAFRS as supplementary cementitious materials in blended cement.

In this research, the negative stereotype of industrial hazardous wastes (e.g., EAFRS) and greenhouse gases (e.g., CO_2_) was converted to the green resources (e.g., supplementary cementitious materials). With the use of EAFRS, significant quantities of CO_2_ in the flue gas can be removed and mineralized as carbonate precipitates via accelerated carbonation. Then, the reacted EAFRS product is further utilized as supplementary cementitious materials in cement mortar. In other words, a waste-to-resource supply chain could be established to achieve a circular economy. The objectives of this study are (1) to determine the performance of flue gas CO_2_ mineralization via accelerated carbonation using EAFRS; (2) to evaluate the improvement on physico-chemical properties of EAFRS after accelerated carbonation; (3) to determine the feasibility of utilizing carbonated EAFRS as green construction materials, including the workability, compressive strength and durability; and (4) to estimate the worldwide CO_2_ reduction potential by mineralization using iron and steel slags.

Via the concept of waste-to-resource supply chain, one ton of EAFRS from steelmaking industry can be used to mineralize 0.38 tons of CO_2_ in the flue gas, corresponding to the carbonation conversion of 83.7%, through an accelerated carbonation process. After carbonation, the reacted EAFRS product is successfully utilized as supplementary cementitious materials, with a substitution ratio of 10%, in cement mortar. Additional benefits from using carbonated EAFRS product in cement mortar, such as reduced leaching potentials of heavy metals, enhanced strength development of mortar, and reduced autoclave expansion, can be realized. We also estimate the global CO_2_ reduction potential by applying CO_2_ mineralization using iron and steel slags, where the amount of annually direct CO_2_ reduction from the flue gas is approximately 0.137 Gt CO_2_.

## Results

### Flue Gas CO_2_ Mineralization via Accelerated Carbonation

**Figure 1:**
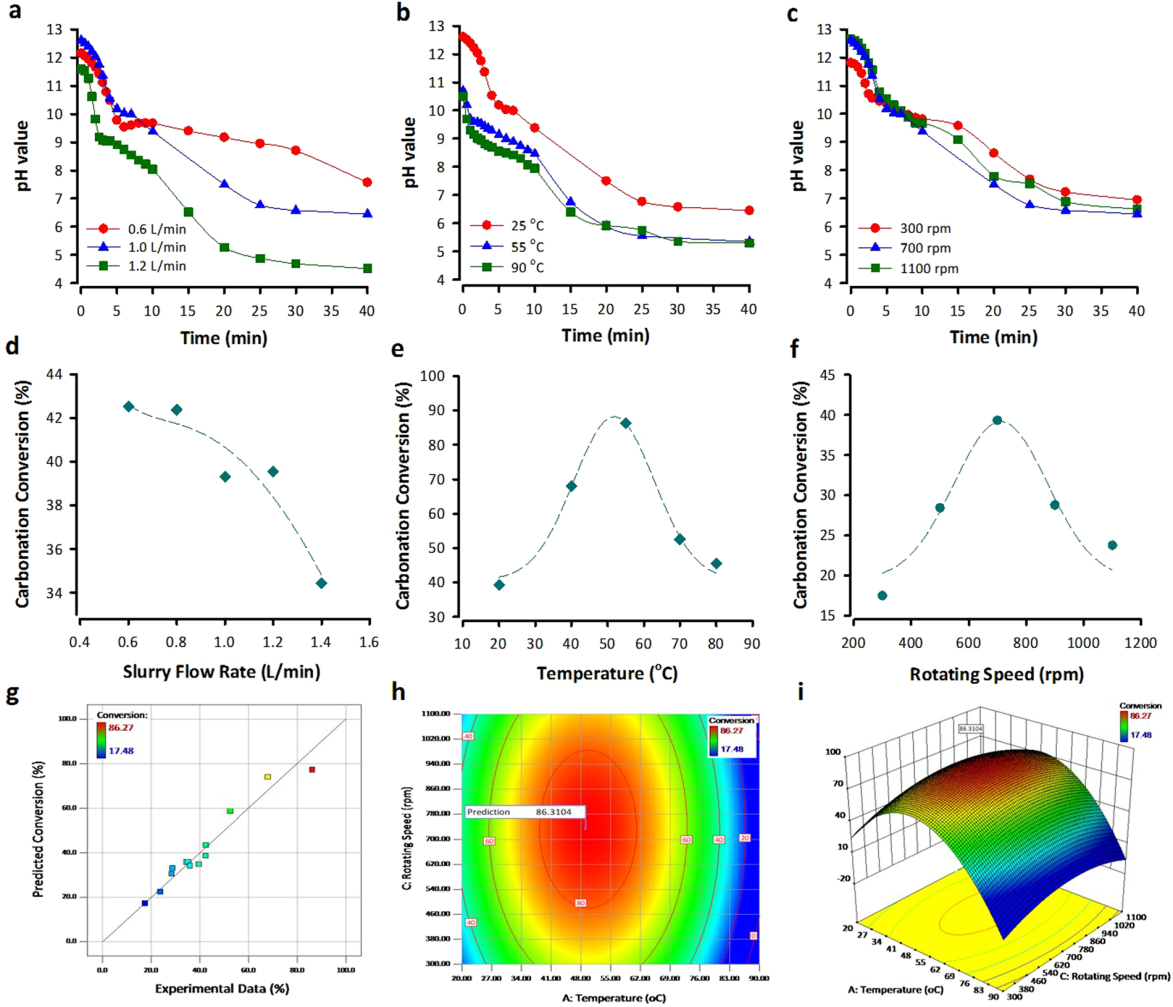
(**a**) Effect of slurry flow rate on pH value, operated at 20 °C and a rotating speed of 700 rpm; (**b**) effect of temperature on pH value, operated at a rotating speed of 700 rpm and a slurry flow rate of 1.0 L/min; (**c**) effect of rotating speed on pH value at 20 °C and a slurry flow rate of 1.0 L/min; (**d**) effect of slurry flow rate on carbonation conversion of EAFRS at 20 °C with an L/S ratio of 25 mL/g and a rotating speed of 700 rpm; (**e**) effect of temperature on carbonation conversion of EAFRS, operated with an L/S ratio of 25 mL/g, a rotating speed of 700 rpm, and a slurry flow rate of 1.0 L/min; (**f**) effect of rotation speed on carbonation conversion of EAFRS at 20 °C with an L/S ratio of 25 mL/g and a slurry flow rate of 1.0 L/min; (**g**) prediction versus actual values of response surface model for carbonation conversion of EAFRS; (**h**) 2D contour and (**I**) 3D response surface plot of carbonation conversion of EAFRS (condition of predicted maximal conversion at a slurry flow rate of 0.6 L/min).

Reaction time (i.e., hydraulic retention time), associated with slurry flow rate and rotating speed, is one of the important design parameters for accelerated carbonation in a rotating packed bed reactor, since it is significantly related to carbonation conversion of alkaline wastes, capacity of CO_2_ mineralization and energy consumption of process. It also affects the pH value of EAFRS slurry along with operation of experiment. Figure 1(a) to (c) show the trends of pH value of EAFRS slurry during a 40-min accelerated carbonation with different operation conditions. The initial pH value of EAFRS slurry was relatively high (i.e., 10.6–12.9), and began to decrease with reaction time after CO_2_ was introduced. After 40-min carbonation, the pH value of EARFS slurry decreased to between 6.5 and 4.5. It is noted that both the dissolution of gaseous CO_2_ and the leaching of calcium ions from solid matrix of EAFRS depend on the pH value. According to Eqs [1] to [3], carbonate (CO_3_^2−^) is the dominant species in the EAFRS slurry when pH is higher than 10.3, and bicarbonate (HCO_3_^−^) is the dominant species in the slurry when pH is between 6.3 to 10.3. Since carbonate is the main species in carbonation reaction, the efficiency of carbonation decreases as the pH value deceases.

1 $${{\rm{CO}}}_{2({\rm{g}})}+{{\rm{H}}}_{{\rm{2}}}{{\rm{O}}}_{({\rm{l}})}\to {{\rm{H}}}_{{\rm{2}}}{{\rm{CO}}}_{{\rm{3}}({\rm{aq}})}^{\ast },\,{{\rm{pK}}}_{{\rm{H}}}=1.5$$

2 $${{\rm{H}}}_{{\rm{2}}}{{\rm{CO}}}_{3(\mathrm{aq})}\to {{\rm{H}}}_{(\mathrm{aq})}^{+}+{{\rm{HCO}}}_{{\rm{3}}\,(\mathrm{aq})}^{-},\,{{\rm{pK}}}_{{\rm{1}}}={\rm{6.3}}$$

3 $${{\rm{HCO}}}_{{\rm{3}}\,(\mathrm{aq})}^{-}\to {{\rm{H}}}_{(\mathrm{aq})}^{+}+{{\rm{CO}}}_{{\rm{3}}\,(\mathrm{aq})}^{2-},\,{{\rm{pK}}}_{{\rm{2}}}=10.3$$

On the other hand, the leaching of calcium ions increases in a low pH value solute, as shown in Eq. [4]:

4 $${\mathrm{Ca}(\mathrm{OH})}_{2({\rm{s}})}\to {{\rm{Ca}}}_{(\mathrm{aq})}^{2+}+{{\rm{2OH}}}_{({\rm{aq}})}^{-},\,{\rm{pK}}={\rm{5.19}}$$

Therefore, the pH value of EAFRS slurry should be carefully controlled to maximize the carbonation efficiency and capacity. The results indicated that the highest carbonation conversion of EAFRS was about 83.7%, corresponding to a mineralization capacity of 0.38 kg CO_2_ per kg EAFRS. Several operating factors, such as slurry flow rate, temperature and rotating speed, play key roles in the performance of accelerated carbonation. For instance, slurry flow rate would influence volumetric mass transfer rate of both liquid- and gas-phase transport phenomena. In general, a higher liquid flow rate would make a thinner liquid film^16^, thereby enhancing the mass transfer between liquid and gas phases based on the two-film theory, as shown in Eq. [5]:

5 $${k}_{CO2}=\frac{{D}_{CO2}}{\delta }$$

where *D*_CO2_ (m^2^/s) is the diffusivity of CO_2_ species which is a function of temperature^17^, *δ* is the film thickness (m), and *k*_CO2_ is the mass transfer coefficient (m/s). According to our previous study^18^, the gas-phase mass transfer coefficient significantly increased (e.g., from 0.094 s^−1^ to 0.366 s^−1^) as the gas-to-slurry ratio decreased (e.g., from 20 to 13, respectively, by increasing slurry flow rate). However, as shown in Fig. 1(d), as the slurry flow rate further increased from 0.6 to 1.4 L/min, the carbonation conversion of EAFRS trend to decrease. This was attributed to the fact that the rapid decrease of pH value under a higher slurry flow rate would restrict carbonation reaction and lead to a lower carbonation conversion of EAFRS. Moreover, a higher slurry flow rate would result in a lower residence time of EAFRS slurry in the reactor, thereby decreasing the carbonation conversion.

Temperature has a significant influence on the carbonation conversion of EAFRS since it is related to (i) the dissolution of reactive oxide from solid waste into solution, (ii) the solubility of CO_2_ into aqueous carbonic species, and (iii) growth and nucleation of carbonate precipitates. For example, under high temperatures, the CO_2_ dissolution from the gas phase into the liquid phase is restricted since the Henry’s constant (*H*) is a function of temperature as described by Eq. [6]^19^:

6 $${H}_{T}={H}_{298K}\times exp[C\times (\frac{1}{T}-1/298)]$$

where *C* is the constant for all gases (2400 K for CO_2_) and *T* is the temperature (K). In other words, the nucleation and growth of CaCO_3_ precipitates were slow due to the solubility of CO_2_ was low which is disadvantageous to carbonation reaction. On the other hand, the leaching of calcium ion from EAFRS increased with the increase of temperature, which is beneficial to carbonation reaction. Therefore, as indicated in Fig. 1(e), a range of optimal temperature from 50–55 °C can be found. In contrast to operating at 20 °C, the calcium ions in EAFRS can be rapidly dissolved into the slurry at 55 °C and compensate for the disadvantage of lower CO_2_ solubility at higher temperatures. While the temperature was over 55 °C the carbonation conversion of EAFRS started decreasing due to the restricted CO_2_ dissolution from the gas phase into the liquid phase. Furthermore, the effect of temperature on pH value was remarkable. The decrease of pH value at high temperatures was much rapid than that at low temperatures.

In addition, since accelerated carbonation is a diffusion control reaction, the effect of rotating speed (i.e., mass transfer rate) on carbonation efficiency should be remarkable. It was observed that the gas-phase mass transfer rate was moderately intensified with an increase of rotor speed^18^. In other words, the mass transfer resistance was reduced as the rotating speed increased. However, a high rotating speed may also cause a reduction in the residence time, resulting in a decrease of carbonation conversion. As shown in Fig. 1(f), the optimal rotating speed for accelerated carbonation of EAFRS should be around 700 rpm. Beyond the optimal point, the contribution of high mass transfer rate would be compensated by the low residence time. In the case of carbonation reaction using basic oxygen furnace slag, the gas-phase mass transfer coefficients varied with the centrifugal acceleration to the 0.33 power^18^, while the liquid-phase mass transfer coefficient was at a scale of 9.23 × 10^−4^ s^−1^ based on the shell mass balance model^20^.

To visualize the carbonation conversion of various operation parameters, the experimental data of accelerated carbonation was further analyzed by the response surface methodology. A D-optimal design was applied to develop a mathematical model of carbonation conversion under various operating conditions, as shown in Eq. [7]:

7 $${\rm{Y}}=77.3-18.0{\rm{A}}-3.8B+2.56C-59.5{A}^{2}+3.9{B}^{2}-15.9{C}^{2}$$

where *Y* is the carbonation conversion of EAFRS; and *A*, *B*, and *C* is independent coded variables of temperature, slurry flow rate, and rotating speed, respectively. Figure 1(g) shows the comparison of the carbonation conversion estimated by the developed model with the experimental data, which indicates that the conversion predicted by the developed model are similar to the experimental value. It was also found that the influence of temperature on carbonation conversion was the most significant (*p* < 0.001). According to the developed model, the maximal conversion of EAFRS should be 86.3% (as shown in Fig. 1(h)), corresponding to a mineralization capacity of 0.394 kg CO_2_ per kg EAFRS, at 49.2 °C with a rotating speed of 733 rpm and a slurry flow rate of 0.6 L/min (as shown in Fig. 1(i)).

It was noted that, in addition to temperature, slurry flow rate, and rotating speed, the CO_2_ concentration in the feed gas stream would affect the accelerated carbonation rate and the CO_2_ fixation capacity of slags^21,22^. In practice, the CO_2_ concentration of the flue gas in the steel industry typically ranges from 10% to 30%. For the sake of comparison, additional experiments of CO_2_ mineralization using EAFRS were operated at a representative CO_2_ concentration of ~30%. With similar operating conditions, the fixation capacity of EAFRS was found to be approximately 0.25 kg CO_2_ per kg of EAFRS, while the corresponding carbonation conversion of EAFRS was about 57.1% with a slurry flow rate of 0.75 L/min at 60 °C.

### Improvement on Physico-Chemical Properties of Alkaline Wastes

**Figure 2:**
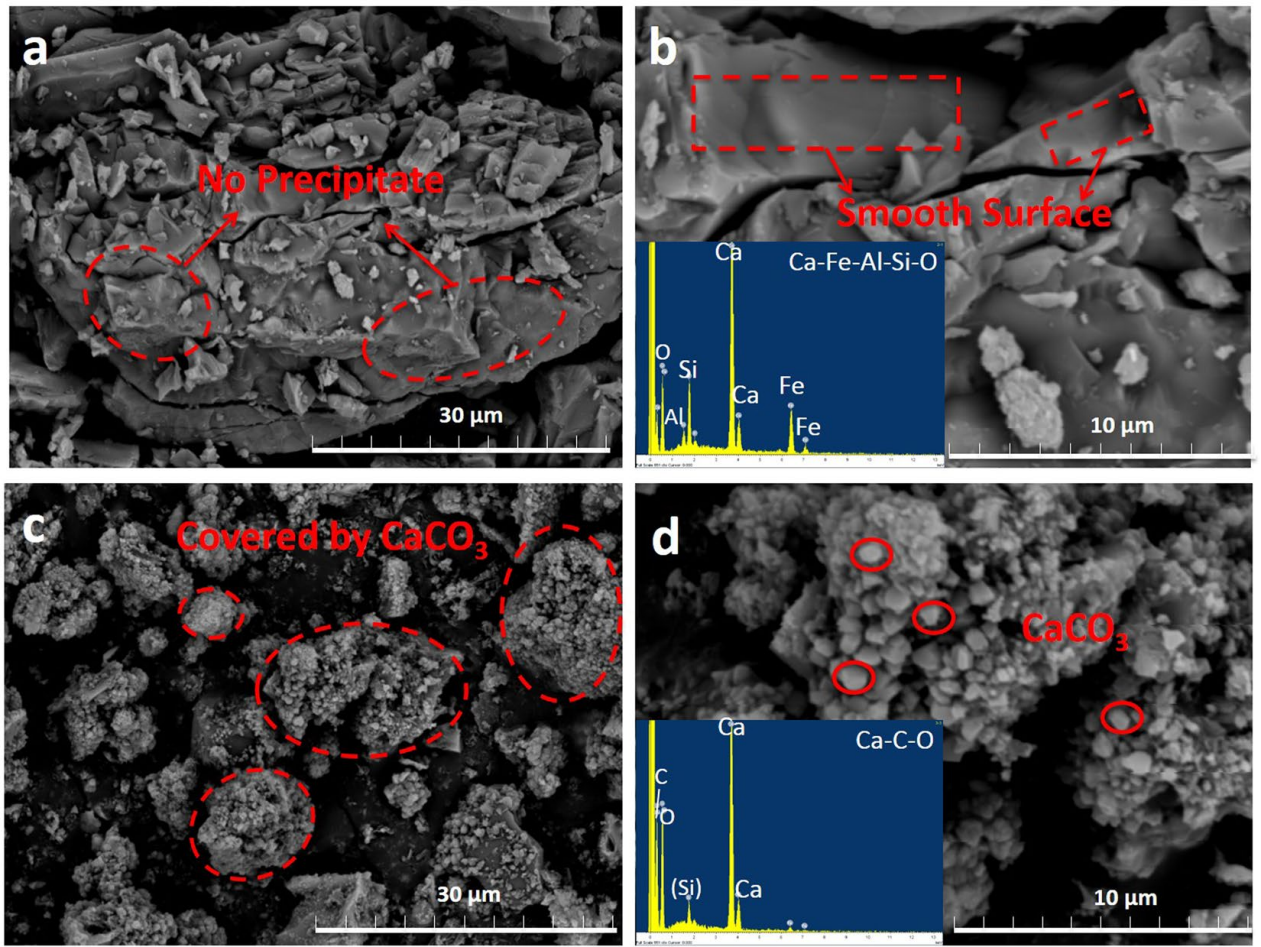
Scanning electron micrographs (SEM) of fresh EAFRS at (**a**) 2500x and (**b**) 8000x associated with EDX analysis; and that of carbonated EAFRS at (**c**) 2500x and (**d**) 8000x associated with EDX analysis.

Accelerated carbonation exhibits the potency of upgrading the physico-chemical properties of alkaline wastes, e.g., high alkalinity and heavy metal leaching. Figure 2 shows the scanning electron microscope (SEM) photos of both the fresh and carbonated EAFRS. Before carbonation, the surface of fresh EAFRS was smooth and flat, as shown in Fig. 2(a) and (b). Compared to the fresh EAFRS, a layer of white material was observed to accumulate on the surface of the carbonated EAFRS, as shown in Fig. 2(c) and (d). The cubic particles with sizes ranging from 1 to 2 μm in diameter were composed primarily of Ca, O, and C (according to the energy-dispersive X-ray spectroscopy (EDX) analyses), which indicated a calcium carbonate composition. The reaction product was also characterized by X-ray diffraction (XRD), as shown in Fig. S1. The main crystal phases of fresh EAFRS were Ca_2_SiO_4_ (calcio-olivine), Ca_2_SiO_5_ (dicalcium silicate), Ca(OH)_2_ (portlandite), and CaO_*f*_ (lime). After carbonation, a significant increase of peak intensities for CaCO_3_ (calcite) was observed, while peaks for both Ca(OH)_2_ and CaO_*f*_ were eliminated. This indicated that the Ca-bearing phases in EAFRS were the main reacting components with CO_2_ to form the carbonate minerals, where the mechanisms can be briefly described as Eqs [8] and [9]:

8 $${\mathrm{Ca}(\mathrm{OH})}_{2({\rm{s}})}+{{\rm{CO}}}_{2({\rm{g}})}\to {{\rm{CaCO}}}_{3({\rm{s}})}+{{\rm{H}}}_{{\rm{2}}}{{\rm{O}}}_{({\rm{l}})}$$

9 $${{\rm{CaO}}}_{{f}({\rm{s}})}+{{\rm{CO}}}_{2({\rm{g}})}\to {{\rm{CaCO}}}_{3({\rm{s}})}$$

After carbonation, the precipitate products (e.g., CaCO_3_) can form a protective layer on the surface of EAFRS, thereby preventing leaching of heavy metals into the solution. Table 1 presents the toxicity characteristic leaching procedure (TCLP) test of fresh and carbonated EAFRS, associated with the limits by regulations in Taiwan. TCLP is an essential indicator for utilizing the carbonated product as construction materials. The results indicate that several metal and non-metal elements in EAFRS can be inhibited from leaching into solution after carbonation. For example, a significant reduction of barium (Ba) ions leaching was achieved after carbonation, e.g., from 0.601 mg/L (in fresh EAFRS) to 0.377 mg/L (in carbonated EAFRS), respectively. The leaching of elements such as selenium (Se) and arsenic (As) with trace leaching concentrations of 0.006 and 0.004 mg/L from fresh EAFRS, respectively, were slightly reduced after carbonation. It thus suggests that the heavy metals in EAFRS can be successfully restricted from leaching into solution via accelerated carbonation. On the other hand, the formed CaCO_3_ protective layer may reduce further diffusion of CO_2_ through the EAFRS, thereby causing the decrease of CO_2_ adsorption ability and carbonation rate. Several studies have been conducted to evaluate the effect of protective product layer on the reaction kinetics^23^. For instance, in the case of accelerated carbonation, since the thickness of product layer was typically much smaller than the particle size of EAFRS, the reduced reaction rate would be neglected^24^.

**Table 1:** Toxicity characteristic leaching procedure (TCLP) results of EAFRS with limits of regulations.

Items^a^	Unit	TCLP test results (solid phase)^b^		Leaching limit (mg/L)		
		Fresh EAFRS (F-EAFRS)	Carbonated EAFRS (C-EAFRS)	Green building materials^c^	Utilization product	Hazardous materials^d^
δ_Cao_	%	0.7	16.0	—	—	—
Hg	mg/L	N.D. (<0.00028)	N.D. (<0.00028)	0.005	0.016	0.2
Pb	mg/L	N.D. (<0.026)	N.D. (<0.026)	0.3	4.0	5.0
Cd	mg/L	N.D. (<0.0027)	N.D. (<0.0027)	0.3	0.8	1.0
Cr (VI)	mg/L	N.D. (<0.0033)	N.D. (<0.0033)	1.5	4.0	2.5
As	mg/L	0.004	0.003	0.3	0.2	5.0
Cu	mg/L	N.D. (<0.010)	N.D. (<0.010)	0.15	0.4	15.0
Cr	mg/L	N.D. (<0.030)	N.D. (<0.030)	N.R.^b^	12.0	5.0
Se	mg/L	0.006	0.004	N.R.^b^	—	1.0
Ba	mg/L	0.601	0.377	N.R.^b^	10.0	100.0

^a^δ_CaO_: carbonation conversion of EAFRS; ^b^C-EAFRS is the carbonated EAFRS for 90 min. N.D.: not detected. N.R.: not regulated. ^c^Green building material standard is regulated by Taiwan architecture and building center; and ^d^Hazardous industrial wastes standard is regulated by NIEA R201.14 C, EPA, Taiwan.

Accelerated carbonation can upgrade the chemical properties of EAFRS, which is beneficial to subsequent utilization as construction materials in blended cement or concrete. Table 2 presents the chemical properties of EAFRS with various carbonation conversions and their associated properties as supplementary cementitious materials with a substitution ratio of 10% in blended cement clinker. The results indicated that, after carbonation, the contents of SiO_2_, MgO and CaO in EAFRS increased while the SO_3_ decreased significantly. Also, no presence of CaO_*f*_ was observed in the carbonated EAFRS, which is beneficial to preventing the cement mortar from expanding in a moist environment. On the other hand, the changes in chemical properties will have an influence on the performance of blended cement. The main components in the ordinary portland cement (OPC) were found to be CaO, SiO_2_, Fe_2_O_3_, Al_2_O_3_ and MgO. It is noted that the major components in carbonated EAFRS are similar to that of OPC, with the percentages of 58.1–58.3% CaO, 17.5–17.6% SiO_2_, 5.4–5.7% Al_2_O_3_, 5.6–6.0% MgO, and 7.8–7.9% Fe_2_O_3_.

**Table 2:** Chemical properties of various carbonation conversions of EAFRS^a^.

Categories	Items	Unit	OPC	F-EAFRS	C-EAFRS-1	C-EAFRS-2	C-EAFRS-3	C-EAFRS-4
Chemical properties	CaO	%	59.70	57.76	58.21	58.06	58.0	58.30
	SiO_2_	%	16.86	15.59	17.59	17.46	17.5	17.50
	Al_2_O_3_	%	5.49	5.19	5.57	5.42	5.66	5.64
	MgO	%	4.44	4.54	5.57	6.03	5.76	5.67
	Fe_2_O_3_	%	8.44	7.80	7.84	7.82	7.87	7.87
	SO_3_	%	3.12	7.05	2.94	2.84	2.87	2.89
	Na_2_O	%	0.58	0.57	0.57	0.53	0.53	0.55
	K_2_O	%	0.62	0.59	0.58	0.56	0.57	0.59
	δ_CaO_	%	—	0.7	1.0	4.0	8.0	16.0
Cement clinker^b^	Initial setting time	min	275	320	272	150	90	75
	Final setting time	min	340	402	350	310	275	275
	Silica ratio	—	—	1.20	1.31	1.32	1.29	1.29
	SO_3_	%	3.12	3.51	3.10	3.09	3.10	3.10

^a^OPC is the ordinary Portland cement. F-EAFRS is the fresh EAFRS. C-EAFRS-1 is the EAFRS after once-through operation. C-EAFRS-2, C-EAFRS-3 and C-EAFRS-4 are the EAFRS carbonated for 30, 60 and 90 min, respectively. ^b^Initial and final setting time of pure OPC mortar, and 10% substitution using fresh or carbonated EAFRS in blended cement mortar.

On the other hand, the effect of carbonation conversion on initial and final setting time of blended cement mortar was remarkable. In general, the addition of supplementary cementitious materials in cement mortar would slightly increase setting time, which is attributed to the cement dilution effect. In this study, the setting time of paste with fresh EAFRS did not increase significantly in comparison with the OPC. In contrast, the setting time of blended cement mortar with carbonated EAFRS decreased as the carbonation conversion increased. This may be attributed that the nucleation sites of EAFRS increases significantly after the carbonation reaction, leading to formation of more hydration products. The well-crystalline CaCO_3_ particles on the surface of carbonated EAFRS can act as seeds for the nucleation of calcium silicate hydrates (CSH), thereby accelerating the formation of the major cement hydration. Additionally, the increased silica ratio after carbonation, to the range of 1.29–1.32, could lead to the reduction in setting time of the mortar. Furthermore, the content of SO_3_ in the cement plays an important role in the compressive strength development. In generally, 2–3% should be the optimum SO_3_ content for cement. Once beyond that (i.e., >4%), compressive strength of cement mortar starts to decline, particularly at early ages. In this study, the content of SO_3_ in cement mortar met the aforementioned criteria.

### Utilization of Carbonated EAFRS as Green Construction Materials

**Figure 3:**
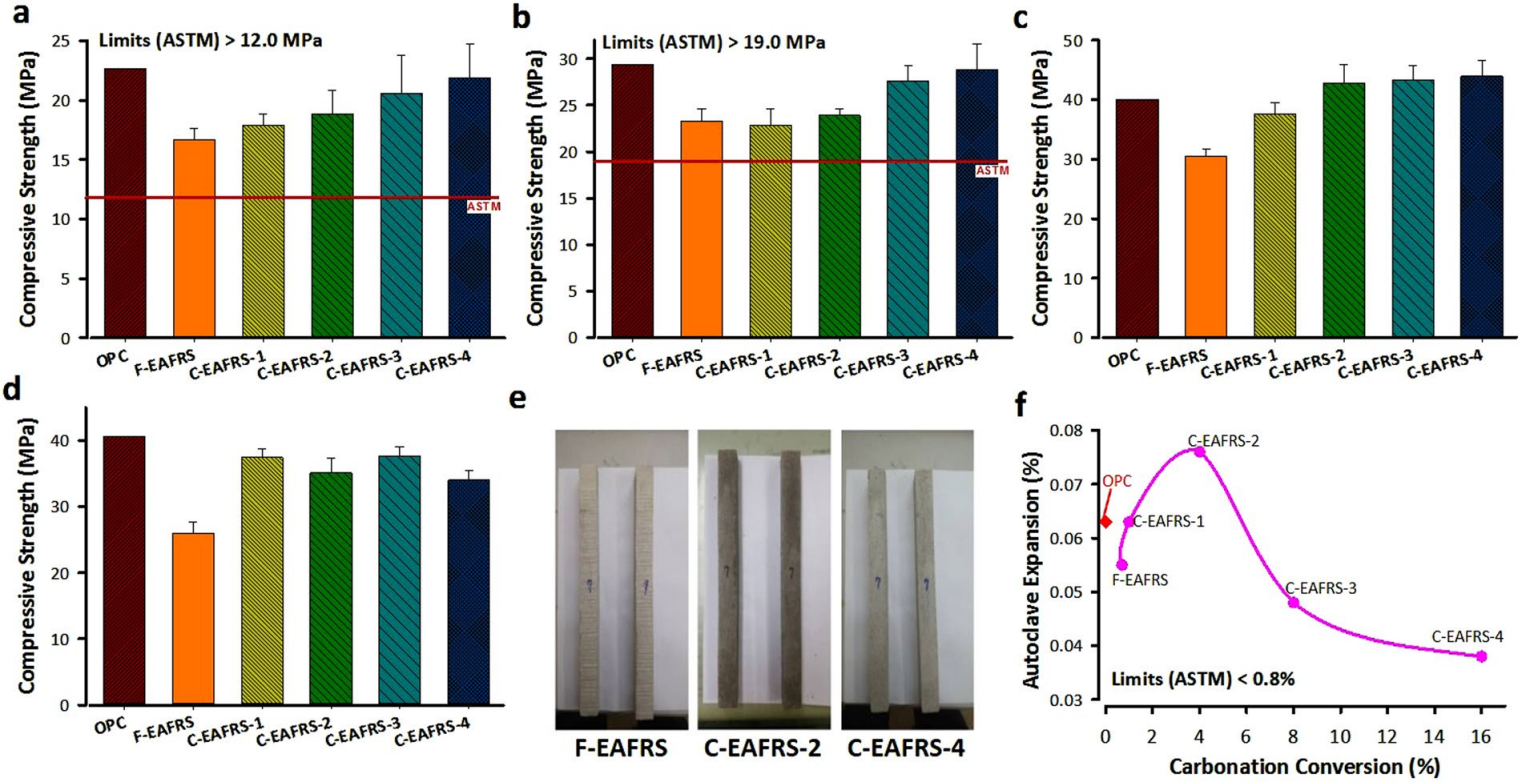
Compressive strength development of various carbonation conversions of EAFRS at (**a**) 3, (**b**) 7, (**c**) 28, and (**d**) 56 days. The ASTM C109/C109M requirements for minimal compressive strength at 3 and 7 d are 12.0 and 19.0 MPa, respectively. (**e**) Photos of autoclave soundness expansion for different cement mortars; (**f**) autoclave soundness expansion of cement mortar using different carbonation conversions of EAFRS.

The performance of utilizing the carbonated EARFS product as supplementary cementitious materials in blended cement mortar, including compressive strength and autoclave expansion, was evaluated, as shown in Fig. 3. In general, the compressive strength increased continuously with the curing age till 28 days, while only a slight increment was observed from 28 to 56 days. The hydration reaction begins after the water is mixed with OPC. As long as the reaction time increases, the hydration product increases as well, thereby enhancing the compressive strength of cement mortar. It is noted that the compressive strength of cement is determined by the mineralogical composition of mortar, such as Ca_3_SiO_5_ (C_3_S), Ca_2_SiO_4_ (C_2_S), Ca_3_Al_2_O_6_ (C_3_A), and Ca_4_Al_2_Fe_2_O_10_ (C_4_AF). For instance, triclinic (C_3_S) contributes to the initial strength development for the early 28 days, while dicalcium silicate (C_2_S) contributes to the ultimate strength for at least one year^25^. Tricalcium aluminate (C_3_A) provides strength for the early stage hydration but few for late stage.

The compressive strengths of all fresh (F-) and carbonated (C-) EAFRS specimen could meet the American Society for Testing and Materials (ASTM) requirements at 3 and 7 days, as shown in Fig. 3(a) and (b), respectively. In general, the C-EAFRS exhibits relatively higher compressive strength than the F-EAFRS, especially at early ages. In the case of C-EAFRS, additional sites for nucleation of the hydration product could be provided due to its high surface area, resulting in a higher compressive strength. It thus suggests that the use of C-EAFRS as supplementary cementitious materials in cement mortar would enhance the mechanical properties to a greater degree than using F-EAFRS.

Compared to blended cement mortar with fresh and carbonated EAFRS substitution, the OPC mortar generally exhibits higher compressive strength at all curing ages. Only at the curing age of 28 days, the blended cement mortar with C-EAFRS (i.e., C-EAFRS-2, C-EAFRS-3 and C-EAFRS-4) obtained higher compressive strength than that with OPC and F-EAFRS, as shown in Fig. 3(c). This might be attributed to the interaction of C_3_A (in OPC) and CaCO_3_ (in C-EAFRS), resulting in the formation of calcium carboaluminate hydrate (C_3_A·CaCO_3_·11 H) and C_3_A·0.5CaCO_3_·0.5Ca(OH)_2_·11.5 H, as shown in Eq. [10]. The calcium carboaluminate hydrate could contribute to a higher mechanical strength.

10 $$\begin{array}{c}{{\rm{2C}}}_{{\rm{3}}}{\rm{A}}+{\rm{1}}{{\rm{.5CaCO}}}_{{\rm{3}}}+{\rm{0}}{\mathrm{.5Ca}(\mathrm{OH})}_{{\rm{2}}}+{\rm{22}}{\rm{.5H}}\to {{\rm{C}}}_{{\rm{3}}}{\rm{A}}\cdot {{\rm{CaCO}}}_{{\rm{3}}}\cdot {\rm{11H}}\\ \quad \quad \,\,+\,{{\rm{C}}}_{{\rm{3}}}{\rm{A}}\cdot {\rm{0}}{{\rm{.5CaCO}}}_{{\rm{3}}}\cdot {\rm{0}}{\mathrm{.5Ca}(\mathrm{OH})}_{{\rm{2}}}\cdot {\rm{11}}{\rm{.5H}}\end{array}$$

where H represents H_2_O. On the other hand, the C_3_A hydration would generate a great amount of hardening heat, which might lead to a negative effect on the compressive strength. The resulted hardening heat would weaken the cement matrix bond with microcrack formation, slightly decreasing the compressive strength on 56 days, as shown in Fig. 3(d). Therefore, there is a trade-off between the initial and ultimate compressive strength, which should be addressed by formulating appropriate ratios of key cementitious compositions (e.g., C_3_S, C_2_S, and C_3_A) with the carbonate products (largely related to the substitution ratios and carbonation conversion of C-EAFRS).

Regarding the volume stability of cement mortar, the autoclave soundness test can provide an index of potential delayed expansion caused by the hydration of free lime (CaO_*f*_), when they present in hydraulic cement. It is noted that the CaO_*f*_ in fresh EAFRS would react with moisture in the air to form Ca(OH)_2_, thereby causing volume expansion. Figure 3(e) and (f) show the specimens and results of autoclave soundness expansion for cement mortars with EAFRS of different carbonation conversions, respectively. The results indicated that the effluence of EAFRS on autoclave expansion was not remarkable. The maximal expansion of blended cement mortar was only 0.076% in the case of C-EAFRS-2. All results of autoclave soundness expansion of cement mortar could reach the ASTM C 150 standard, i.e., less than 0.8%.

### Potency of Worldwide CO_2_ Reduction

**Figure 4:**
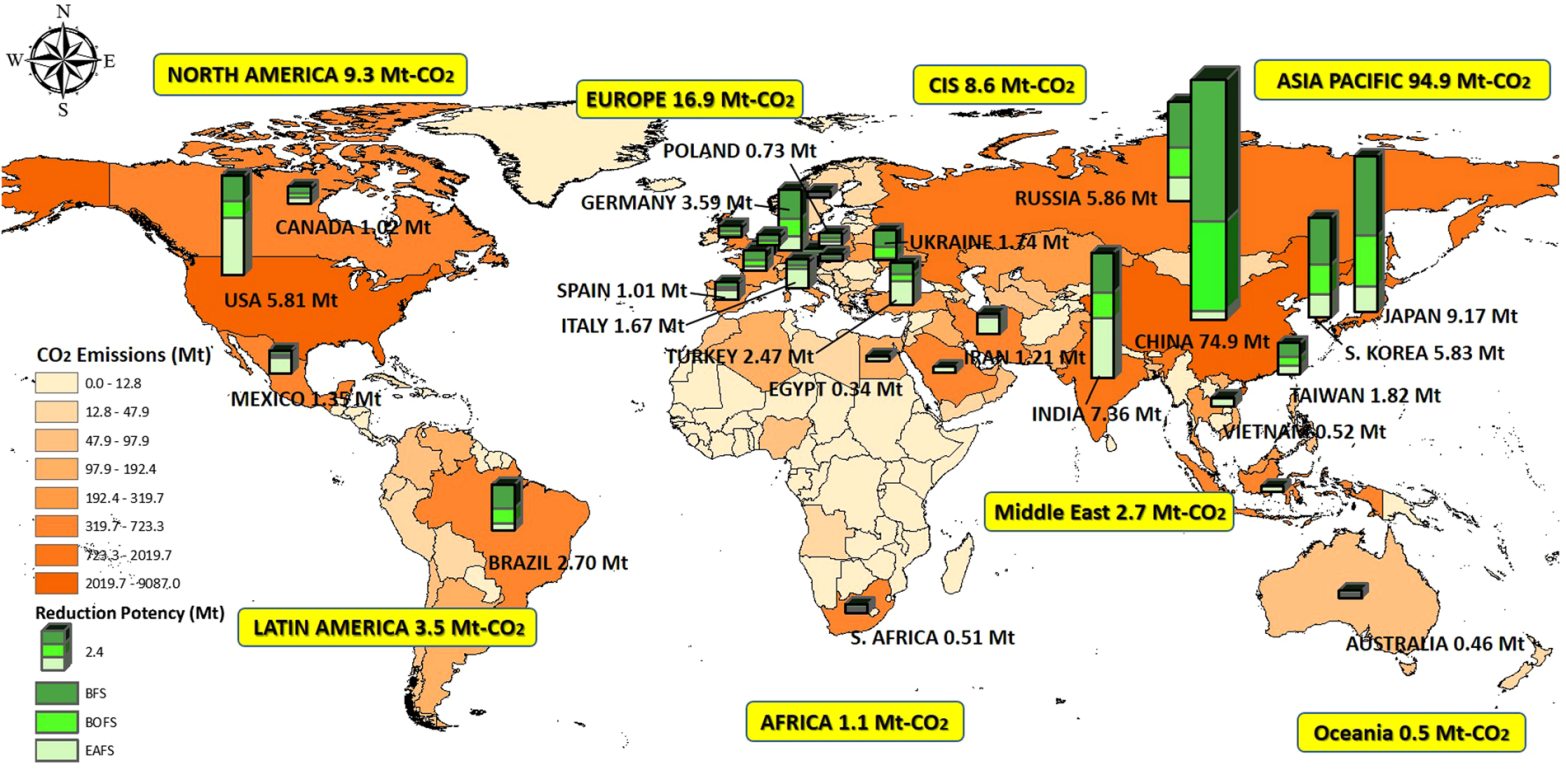
Potency of direct CO_2_ reduction by establishing waste-to-resource supply chain exemplified by iron and steel slags including blast furnace slag (BFS), basic oxygen furnace slag (BOFS), and electric arc furnace slag (EAFS). The maps were generated using Geographic Information Systems software ArcGIS ver. 10.2^29^ (http://www.esri.com/). The World spatial boundary GIS data is from the Open Database of Global Administrative Areas (GADM) ver. 2.0^30^ (http://www.gadm.org/), which provides the basic GIS data for the World Imagery.

A district green supply chain can be established by considering the iron and steel industry as a district supply center of green resources. The worldwide crude steel production in 2016 was approximately 1628 Mt, where the percentages of oxygen furnace and electric furnace were 74.3% and 25.3%, respectively^26^. In other words, significant quantities of iron and steel slags will be generated along the steelmaking process. Figure 4 estimates the worldwide potency of annual direct CO_2_ reduction by establishing the waste-to-resource supply chain exemplified by iron and steel slags. The amount of worldwide reduction by CO_2_ mineralization using iron and steel slags was approximately 137.5 Mt per year, contributing to a reduction of the global anthropogenic CO_2_ emissions (i.e., ~36 Gt of CO_2_ per year) by 0.38%. The Asia Pacific area exhibits the greatest potential of direct CO_2_ reduction, i.e., around 94.9 Mt of CO_2_ per year, followed by the Europe (i.e., about 16.9 Mt of CO_2_ per year). In particular, China possesses the largest annual CO_2_ reduction potential of 74.9 Mt, although it also contributes a great amount of anthropogenic CO_2_ emissions annually. The second large potential of CO_2_ reduction is Japan at about 9.2 Mt CO_2_ per year.

## Discussion

The chemical compositions of EAFRS largely affect the performance of CO_2_ mineralization, as well as the subsequent utilization as supplementary cementitious materials in cement mortar. For CO_2_ fixation, both Ca- and Mg-bearing species are usually considered as the major reactive components to form carbonate precipitates. Typical reaction temperature and CO_2_ pressure for the formation of MgCO_3_ via aqueous carbonation should be higher than 144 °C and 100 kg/cm^2^, respectively^27^. Therefore, in this study, the potency of MgCO_3_ formation could be neglected due to its relatively low operating temperature and pressure of CO_2_. Other components in EAFRS, such as SiO_2_, Al_2_O_3_, Fe_2_O_3_, and SO_3_, do not contribute to CO_2_ fixation. However, for utilization of EAFRS in cement mortar, the content of SiO_2_ in EAFRS would contribute to pozzolanic activity of cement mortars, which is highly related to strength development. Similarly, Al_2_O_3_ may cause the change in viscosity of blended cement or concrete pastes, affecting their self-leveling and pumping behavior, as well as related to solid particles segregation.

**Figure 5:**
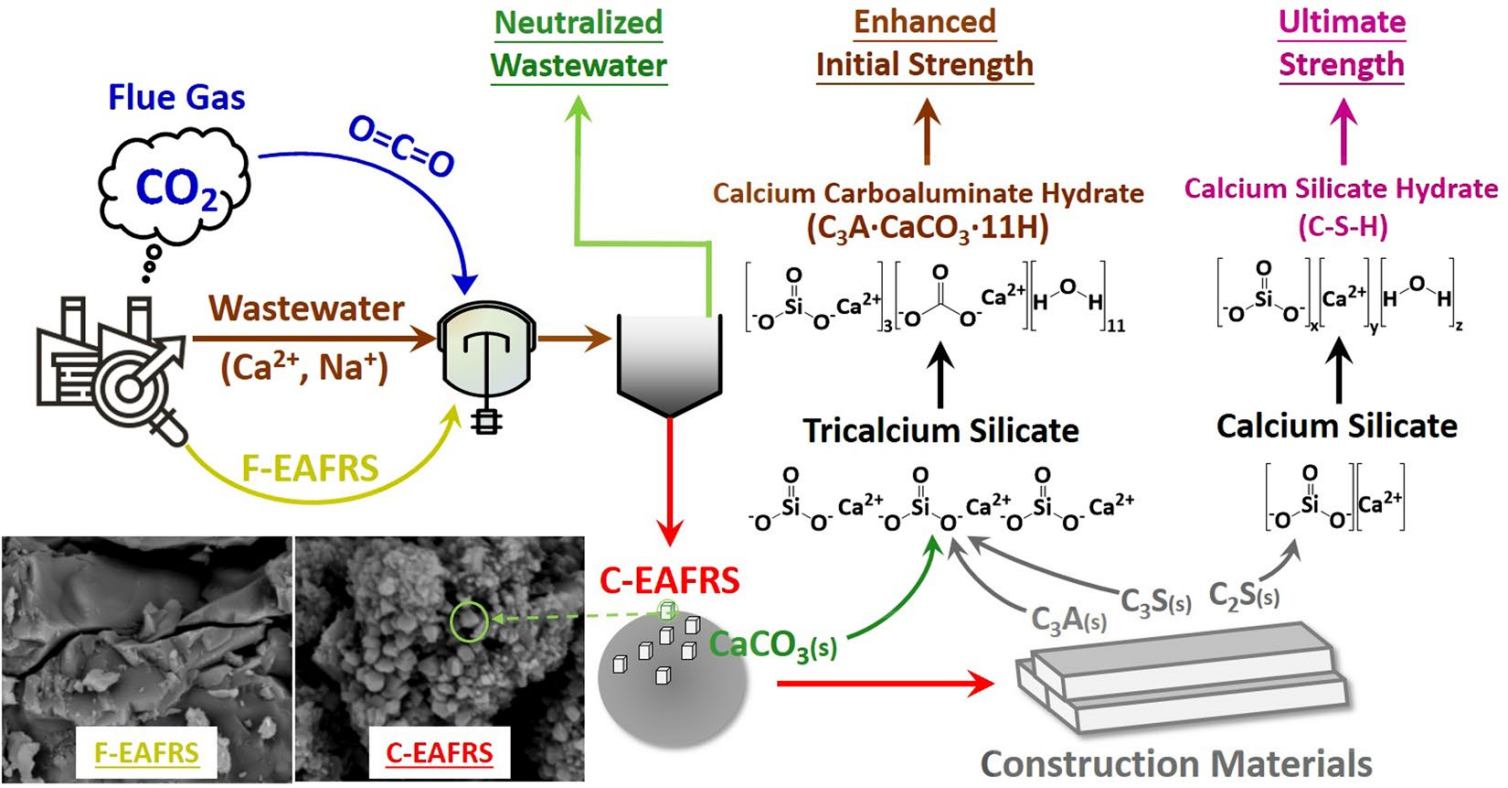
Scheme of waste-to-resource supply chain for CO_2_ mineralization and utilization exemplified by electric arc furnace reducing slag.

In this study, we report an integrated approach to mineralizing CO_2_ in the flue gas as carbonate precipitates via accelerated carbonation, while utilizing the reacted product as supplementary cementitious materials in cement mortar. Under this framework, a waste-to-resource supply chain could be established toward a circular economy, as shown in Fig. 5. The maximum carbonation conversion of 86.3%, corresponding to a capacity of 0.38 t-CO_2_ per ton of EAFRS, can be obtained at 55 °C. In addition, the use of carbonated EAFRS as supplementary cementitious materials in cement mortars exhibited superior performance on the workability, mechanical strength development, and durability to that of using fresh EAFRS. Moreover, the leaching potentials for several heavy metals (e.g., Ba, Se, and As) from EAFRS were significantly reduced after carbonation.

However, the amounts of CO_2_ fixation by the mineralization process could be offset by the energy usage of the process (e.g., grinding unit, reactor, agitator, slurry pump and heater) since electricity generation may cause additional CO_2_ emissions. Therefore, the net CO_2_ fixation amounts of the entire waste-to-supply chain should be critically evaluated by the life cycle assessment. It is worth mentioning that cement production is a CO_2_-intensive process, in which roughly 0.73–1.00 tons of CO_2_ are generated for one ton of cement production^28^. By utilization of the carbonated EAFRS as supplementary cementitious materials, a significant amount of CO_2_ emission could be indirectly avoided. This would contribute more to the reduction of total CO_2_ emissions than mineralization. Thus, increasing the substitution ratio of reacted EAFRS in cement mortar while meeting the ASTM requirement for essential characteristics, functions and qualities became more critical to maximize the overall CO_2_ mitigation capacity.

From the perspective of material flow, a huge amount of water is needed to enhance the reaction kinetics of accelerated carbonation. On the other hand, freshwater stress and scarcity are two of the most challenging issues caused by climate change, rapid population growth, and urbanization. Therefore, utilizing industrial wastewater should be a resource-efficient approach to CO_2_ minimization via accelerated carbonation, especially for the industries in water-scarce regions. It is noted that the chemical properties of industrial wastewater can be upgraded after accelerated carbonation via several mechanisms, such as neutralization and precipitation. In this work, the pH value of EARFS slurry was found to decrease to 4.5–6.5 after 40-min carbonation.

To achieve a circular economy, the utilization of industrial solid wastes should be considered as one of the available demonstration portfolio for establishing the waste-to-resource supply chain. It is noted that the worldwide CO_2_ reduction by mineralization using iron and steel slags (only) is approximately 0.137 Gt per year, corresponding to a global CO_2_ emission reduction by 0.38%. In addition to iron and steel slags, there are numerous industrial alkaline solid wastes, such as cement kiln dust, municipal solid waste incinerator ashes, air pollution control ashes, shell ashes and sludge, that can be used for CO_2_ mineralization and utilization. On the other hand, the reacted product can be used as substitution materials in cement/concrete, which can further contribute to a more significant amount of indirect CO_2_ reduction. Therefore, the overall potential of CO_2_ reduction should be critically assessed by considering both the direct portions (via the mineralization process) and indirect avoidance (due to the product utilization) of CO_2_ reduction. Future research should focus on technology evaluation, including life cycle assessment, techno-economic analysis, and waste-water-energy nexus, for system optimization.

## Methods

### Materials

In this study, EAFRS was provided by Tung Ho Steel Enterprise Corporation, Taiwan. The EAFRS was crushed into smaller debris and then milled into fine particles by a ball-mill machine. Afterwards, the EAFRS was sieved to a particle size less than 15 µm and then dried in an oven at 105 °C overnight to eliminate moisture. The components in EAFRS include CaO (56.4%), SiO_2_ (26.6%), Al_2_O_3_ (6.3%), MgO (5.3%), Fe_2_O_3_ (2.0%), SO_3_ (1.1%), and free lime (CaO_*f*_, 0.8%). For the CO_2_ mineralization experiments, de-ionized (DI) water with 18.2 MΩ/cm was used to form the EAFRS slurry. High-pressure CO_2_ and N_2_ gases (with a purity of 99.5%) were purchased from Ching-Feng Gas Corporation, Taiwan. For the product utilization experiments, the physico-chemical properties of OPC were checked in compliance with ASTM C150 specifications. Graded standard sand (Ottawa sand) following ASTM C778 was used to prepare mortar specimens.

### CO_2_ Mineralization through Accelerated Carbonation

In this study, CO_2_ mineralization through accelerated carbonation of EAFRS was conducted in a rotating packed bed reactor, known as a high-gravity carbonation process. It was noted that the carbonation conversion of basic oxygen furnace slag can be significantly improved within a short reaction time at ambient temperatures and pressure^12^. Stainless steel wire was used as a packing material in the rotating packed bed reactor. The reactor, with an inner and outer radius of 0.077 and 0.165 m, respectively, can be operated at a rotating speed up to 1,800 rpm. The packing zone had an axial height of 0.020 m.

The EAFRS is well-mixed with DI water to form EAFRS slurry at a liquid-to-solid (L/S) ratio of 25 mL/g. When the experiment begins, CO_2_ gas flows into the reactor, and then carbonation reaction begins; at the same time, the slurry will pump into the reactor for further reaction. The gas flow rate was 2.5 L/min with a CO_2_ concentration of 99.9 vol% at a pressure of 1.03 kg/cm^2^. The ranges of variable parameters for temperature, EAFRS slurry flow rate and rotating speed were 25–80 °C, 0.6–1.5 L/min, and 300–1,100 rpm, respectively. The samples were taken at 1, 2, 3, 4, 5, 7.5, 10, 15, 20, 30 and 40 mins, and then dried in an oven at 105 °C overnight to eliminate moisture. Afterward, the carbonation conversion of EAFRS samples was analyzed by thermogravimetric analysis (TGA).

### Preparation of Cement Mortar with EAFRS

In this study, blended cement mortars were prepared by mixing with fresh or carbonated EAFRS at a substitution ratio of 10%. Different carbonation conversions of EAFRS (i.e., 0.7%, 1.0%, 4.0%, 8.0%, and 16.0%) were blended in cement mortar for evaluating the performance of compressive strength and autoclave expansion. To maintain consistent workability in cement pastes, a standard flow of 110 ± 5%, as specified by ASTM C230, was maintained by adjusting the quantity of mixing water in a blended cement mortar. For evaluation of compressive strength, the standard sand was mixed with cement and EAFRS to become a paste, and then put into cubic molds with a size of 5 mm × 5 mm × 5 mm. The molds were cured for 24 h to set the specimens, and then the cubic specimens were demolded afterward. The cubic specimens were cured in a saturated lime solution at room temperature for time intervals of 3, 7, 28, and 56 days, which were used for compressive strength testing.

### Performance Evaluation of Blended Cement Mortars

The determination of setting time for cement blended was in accordance with ASTM C191 using a Vicat needle. The initial and final setting times should be no less than 45 min and no more than 7 h, respectively. Compressive strength is one of the most important mechanical property of blended cement mortar. The ASTM C109/C109M for minimal compressive strength at 3 and 7 days are 12.0 and 19.0 MPa, respectively (i.e., 1760 and 2760 psi, respectively). In this study, the mass ratio of sand, OPC and supplementary cementitious materials in blended cement mortar was 2.75: 0.90: 0.10, and all blended cement mortars were cured in a saturated lime solution for 56 days. For the control group, a cube paste with 100% OPC type I (i.e., no replacement using EAFRS) was prepared. On the other hand, autoclave expansion is one of the useful indicators for the stability of construction materials. The ASTM C151 requirement for portland cement specifies a maximum autoclave expansion of 0.80%. In the autoclave expansion tests, cement paste bars were first cured at room temperature for 24 h, and then placed in an autoclave equipment with the steam pressure of 2.00 ± 0.07 MPa at 216 ± 2 °C for 3 h.

### Analytical Techniques

After CO_2_ mineralization experiments, the sampled EAFRS slurry was treated by vacuum filtration and then dried in an oven to 105 °C. Then, TGA (STA6000, PerkinElmer) was used to determine the carbonation conversion of EAFRS. The EAFRS sample was heated directly from 50 to 950 °C at a heating rate of 10 °C per min. The chemical composition, mineralogy and microstructure of EAFRS before and after carbonation were evaluated by XRF (PW2430, Phillips), XRD (Bruker D8), and SEM (JSM-4500, JEOL), respectively. The distributions of elements on the surface of fresh and carbonated EAFRS were detected using energy-dispersive X-ray (EDX) spectroscopy. The EDX can be employed for characterizing the formed CaCO_3_ during the carbonation reaction. In addition, the toxicity characteristic leaching procedure (TCLP) for fresh and carbonated EAFRS was carried out. The leaching limits for hazardous industrial wastes, specified by NIEA R201.14 C, as well as green building material, regulated by the Taiwan Architecture & Building Center, were used to determine if the product could be utilized as a construction material.

### Estimate of Worldwide CO_2_ Reduction Potency

World maps of CO_2_ emissions, as well as the CO_2_ reduction potential contributed by carbon mineralization of steel slags were compiled using Geographic Information Systems (GIS) software ArcGIS (v. 10.2; http://www.esri.com/)^29^. The World spatial boundary GIS data is from the Open Database of GADM (v. 2.0; http://www.gadm.org/)^30^. The International Energy Agency (IEA) provided CO_2_ emissions by regions and countries^31^. The information of worldwide production of crude steel, as well as oxygen furnace and electric furnace slags in 2016 was gathered from the World Steel Association^26^. The CO_2_ reduction potential contributed by carbon mineralization of steel slags, including blast furnace slag, basic oxygen furnace slag and electric arc furnace slag, was estimated by multiplying their annual production with the maximum achievable carbonation conversion obtained in this study and those in the literature^7^.

### Data Statement

Readers can access the data via contact to the authors.

## Electronic supplementary material

Supplementary information

## References

[1] Matter JM (2016). Rapid carbon mineralization for permanent disposal of anthropogenic carbon dioxide emissions. Science..

[2] Markewitz P (2012). Worldwide innovations in the development of carbon capture technologies and the utilization of CO_2_. Energy & Environmental Science..

[3] Mac Dowell N, Fennell PS, Shah N, Maitland GC (2017). The role of CO_2_ capture and utilization in mitigating climate change. Nature Clim. Change..

[4] Kirchofer A, Brandt A, Krevor S, Prigiobbe V, Wilcox J (2012). Impact of alkalinity sources on the life-cycle energy efficiency of mineral carbonation technologies. Energy & Environmental Science..

[5] Gao Y (2017). BOF steel slag as a low-cost sorbent for vanadium (V) removal from soil washing effluent. Sci Rep..

[6] World Steel Association. Steel industry by-products. 2 (2016).

[7] Pan SY (2015). High-Gravity Carbonation Process for Enhancing CO_2_ Fixation and Utilization Exemplified by the Steelmaking Industry. Environ Sci Technol..

[8] Bourzac K, Savage N, Owens B, Scott AR (2017). Materials and engineering: Rebuilding the world. Nature..

[9] Pan S-Y, Adhikari R, Chen Y-H, Li P, Chiang P-C (2016). Integrated and innovative steel slag utilization for iron reclamation, green material production and CO_2_ fixation via accelerated carbonation. Journal of Cleaner Production..

[10] Sanna A, Uibu M, Caramanna G, Kuusik R, Maroto-Valer MM (2014). A review of mineral carbonation technologies to sequester CO_2_. Chemical Society reviews..

[11] Lackner KS (2003). Climate change. A guide to CO_2_ sequestration. Science..

[12] Pan SY, Chiang PC, Chen YH, Tan CS, Chang EE (2013). *Ex Situ* CO_2_ capture by carbonation of steelmaking slag coupled with metalworking wastewater in a rotating packed bed. Environ Sci Technol..

[13] Pan SY (2013). Systematic Approach to Determination of Maximum Achievable Capture Capacity via Leaching and Carbonation Processes for Alkaline Steelmaking Wastes in a Rotating Packed Bed. Environ Sci Technol..

[14] Mikkelsen M, Jørgensen M, Krebs FC (2010). The teraton challenge. A review of fixation and transformation of carbon dioxide. Energy & Environmental Science..

[15] Tomkinson T, Lee MR, Mark DF, Smith CL (2013). Sequestration of Martian CO_2_ by mineral carbonation. Nat Commun..

[16] Liu H, Kuo C (1996). Quantitative multiphase determination using the Rietveld method with high accuracy. Materials Letters..

[17] Chiang, P.-C. & Pan, S.-Y. In *Carbon Dioxide Mineralization and Utilization* Ch. 7, (Springer Nature Singapore Pte Ltd., 2017).

[18] Pan SY (2015). Systematic approach to determination of optimum gas-phase mass transfer rate for high-gravity carbonation process of steelmaking slags in a rotating packed bed. Applied Energy..

[19] Morel, F. M. & Hering, J. G. *Principles and applications of aquatic chemistry*. (John Wiley & Sons, 1993).

[20] Chang EE, Chen T-L, Pan S-Y, Chen Y-H, Chiang P-C (2013). Kinetic modeling on CO_2_ capture using basic oxygen furnace slag coupled with cold-rolling wastewater in a rotating packed bed. J Hazard Mater..

[21] Chang EE (2015). Accelerated carbonation using municipal solid waste incinerator bottom ash and cold-rolling wastewater: Performance evaluation and reaction kinetics. Waste Manag..

[22] Chang EE, Wang Y-C, Pan S-Y, Chen Y-H, Chiang P-C (2012). CO_2_ Capture by Using Blended Hydraulic Slag Cement via a Slurry Reactor. Aerosol and Air Quality Research..

[23] Sohn HY (2004). The effects of reactant starvation and mass transfer in the rate measurement of fluid–solid reactions with small equilibrium constants. Chemical Engineering Science..

[24] Chang EE, Pan SY, Chen YH, Tan CS, Chiang PC (2012). Accelerated carbonation of steelmaking slags in a high-gravity rotating packed bed. J Hazard Mater..

[25] Pan S-Y (2016). Integrated CO_2_*Fixation, Waste Stabilization, and Product* Utilization via High-Gravity Carbonation Process Exemplified by Circular Fluidized Bed Fly Ash. ACS Sustainable Chemistry & Engineering..

[26] World Steel Association. *World Steel in Figures 2017*. 10 (World Steel Association, 2017).

[27] Teir, S. *Fixation of carbon dioxide by producing carbonates from minerals and steelmaking slags* Doctoral Dissertation thesis, Helsinki University of Technology, (2008).

[28] Hasanbeigi A, Price L, Lin E (2012). Emerging energy-efficiency and CO_2_ emission-reduction technologies for cement and concrete production: A technical review. Renewable and Sustainable Energy Reviews..

[29] ArcGIS. (ed Environmental Systems Research Institute) (http://www.esri.com/legal/copyright-trademarks, Redlands, CA, 2011).

[30] GADM. (ed Global Administrative Areas) (GADM database of Global Administrative Areas, http://www.gadm.org/, 2012).

[31] IEA. Key World Energy Statistics. 100 (OECD, International Energy Agency, France, 2017).

