# Supplementary material for: CO2 Mineralization and Utilization using Steel Slag for Establishing a Waste-to-Resource Supply Chain
Source: Sci Rep. 2017 Dec 8;7:17227. doi: 10.1038/s41598-017-17648-9 (PMC5722910; doi:10.1038/s41598-017-17648-9)
Supplement: Supplementary file 1 — Supplementary information [file 41598_2017_17648_MOESM1_ESM.doc]

**CO_2_ Mineralization and Utilization using Steel Slag for Establishing a Waste-to-Resource Supply Chain**

Shu-Yuan Pan^1,2^, Tai-Chun Chung^1^, Chang-Ching Ho^3^, Chin-Jen Hou^3^, Yi-Hung Chen^4^, & Pen-Chi Chiang^1,2^

^1^ Graduate Institute of Environmental Engineering, National Taiwan University, Taipei, 10673 Taiwan

^2^ Carbon Cycle Research Center, National Taiwan University, Taipei, 10674 Taiwan

^3^ Tung Ho Steel Enterprise Corporation, Miaoli, 368 Taiwan

^4^ Department of Chemical Engineering and Biotechnology, National Taipei University of Technology, Taipei, 10608 Taiwan

* Correspondence and requests for materials should be addressed to S.Y.P. (email: d00541004@ntu.edu.tw) or P.C.C. (email: [pcchiang@ntu.edu.tw](mailto:pcchiang@ntu.edu.tw))

**Supplementary legends:**

**Figure S1.** X-ray diffraction (XRD) patterns of fresh (F-) and carbonated (C-) EAFRS.

**Figure S1.** X-ray diffraction (XRD) patterns of fresh (F-) and carbonated (C-) EAFRS.

**
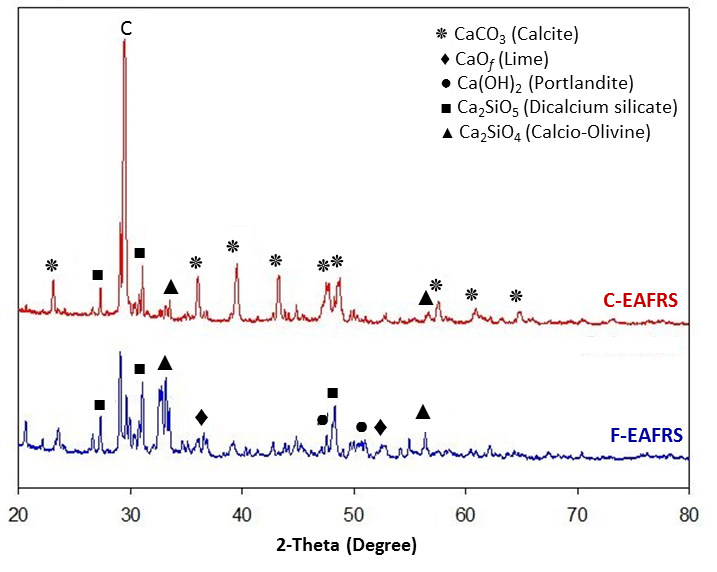
**
